# Comparison of different approaches to estimating age standardized net survival

**DOI:** 10.1186/s12874-015-0057-3

**Published:** 2015-08-15

**Authors:** Paul C. Lambert, Paul W. Dickman, Mark J. Rutherford

**Affiliations:** Department of Health Sciences, University of Leicester, University Road, Leicester, LE1 7RH UK; Department of Medical Epidemiology and Biostatistics, Karolinska Insitutet, Stockholm, PO BOX 281, 24105 Sweden

**Keywords:** Net survival, Relative survival, Epidemiology

## Abstract

**Background:**

Age-standardized net survival provides an important population-based summary of cancer survival that appropriately accounts for differences in other-cause mortality rates and standardizes the population age distribution to allow fair comparisons. Recently, there has been debate over the most appropriate method for estimating this quantity, with the traditional Ederer II approach being shown to have potential bias.

**Methods:**

We compare lifetable-based estimates (Ederer II), a new unbiased method based on inverse probability of censoring weights (Pohar Perme) and model-based estimates. We make the comparison in a simulation setting; generating scenarios where we would expect to see a large theoretical bias.

**Results:**

Our simulations demonstrate that even in relatively extreme scenarios there is negligible bias in age-standardized net survival when using the age-standardized Ederer II method, modelling with continuous age or using the Pohar Perme method. However, both the Ederer II and modelling approaches have some advantages over the Pohar Perme method in terms of greater precision, particularly for longer-term follow-up (10 and 15 years).

**Conclusions:**

Our results show that, when age-standardizing, concern over bias with the traditional methods is unfounded. We have also shown advantages in using the more traditional and modelling methods.

**Electronic supplementary material:**

The online version of this article (doi:10.1186/s12874-015-0057-3) contains supplementary material, which is available to authorized users.

## Background

Cancer survival is often compared between different populations. For example, between regions [1], socio-economic groups [2] or calendar periods [3]. There are many studies that compare cancer survival between countries, for example the EUROCARE [4], CONCORD [5] and International Cancer Benchmarking Partnership [6] studies.

Comparison of cancer survival is complicated by mortality due to other causes varying between the groups being compared. This will impact on the probability of actually dying of the cancer. Therefore comparisons of cancer survival between populations usually attempt to estimate *net survival*, which is interpreted as survival in the hypothetical world where it is not possible to die from other causes [7]. This allows a ‘fair’ comparison of cancer survival. Net survival is something we never observe as patients in the real world are at risk of death from other causes. Therefore, estimation requires assumptions to be made. The usual approach has been to use *relative survival* to estimate net survival.

Age plays an important role in the comparison of cancer survival. For most cancers net survival decreases with age. When comparing survival between populations it is therefore essential to ‘adjust’ for differences in the age distribution. When a single summary measure is required, traditional age standardization is used, where an estimate of relative survival is obtained separately in age groups and a weighted average calculated with the weights reflecting an international standard population [8].

Often survival differences between groups vary by age. For example, international comparisons for breast [9, 10], colorectal [11], prostate [12] and lung cancer [13] showed larger differences in relative survival with increasing age. By just giving an overall average these important differences can be missed. However, age-standardized relative survival is still an important summary measure and will continue to be used.

Net survival has traditionally been estimated using relative survival; the ratio of all-cause survival to expected survival. Pohar Perme *et al.* showed the commonly used Ederer II method and other methods are potentially biased when interest lies in an overall average of net survival and suggest an alternative (the Pohar Perme method) [14]. There has been inconsistency in the definition of ‘relative survival’ which has been used to refer to an analysis where all ages have been pooled together [14, 15]. Similarly, the term ‘net survival’ has been used to describe the average net survival in a population. However, the concepts of net and relative survival exist at the individual level. In fact we argue that the Pohar-Perme method is estimated in a relative survival framework and can only be interpreted as average net survival under two key assumptions that apply to all methods.

An alternative way to estimate net survival is to use statistical modelling [16, 17]. Statistical modelling allows various assumptions to be incorporated, for example proportional excess hazards, only selected interactions or the fact that the true net survival can never increase.

**Fig. 1:**
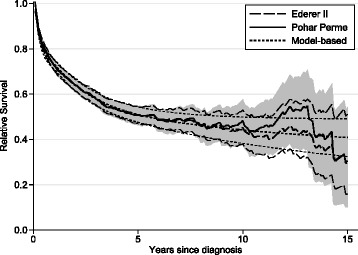
Comparison of Different Approaches to Estimating Net Survival (with 95 % confidence intervals) for 2117 Men Aged 75+, Diagnosed with Cancer of the Rectum in England between 1992 and 2007. The Confidence Interval for the Pohar Perme Method is Shown by the Shaded Area

Figure 1 shows estimated net survival for cancer of rectum in England for those age 75+ estimated using three different methods described later (Ederer II, Pohar Perme and model based). It illustrates three key issues,(i) the methods give different estimates of net survival, particularly for long term follow up; (ii) the Pohar-Pohar estimate is more variable than the other methods; (iii) the confidence intervals for the Pohar-Perme estimate are wider.

We argue that the theoretical bias when using the Ederer II method to estimate age group specific or age-standardized net survival is negligible and can effectively be ignored in practice. However, there are some advantages in terms of improved precision when using Ederer II or statistical modelling over the Pohar Perme method.

## Methods

### Excess mortality, relative survival and net survival

We explain key concepts through both the excess mortality rate and relative survival as this highlights differences between the methods. The excess mortality rate for an individual *i* is the difference between their all-cause mortality rate, *h*_*i*_(*t*) and their expected mortality rate if they did not have cancer, $h_{i}^{*}(t)$,

$$\lambda_{i}(t) = h_{i}(t) - h_{i}^{*}(t). $$

The subscripts, *i*, are important as both expected and all-cause mortality varies between individuals, with age a key factor. Expected mortality rates are usually obtained from national or regional mortality statistics, stratified by age, sex, calendar year and potentially other covariates.

The relative survival function, *R*_*i*_(*t*) for an individual, *i*, is

(1) $$  R_{i}(t) = \exp\left(-{\int_{0}^{t}} \lambda_{i}(u) du\right) = \frac{S_{i}(t)}{S_{i}^{*}(t)}  $$

where *S*_*i*_(*t*) and $S_{i}^{*}(t)$ are the all-cause survival and expected survival functions. To interpret, *R*_*i*_(*t*) as a net probability of survival, ${S_{i}^{N}}(t)$, i.e. in the hypothetical situation where it is not possible to die of other causes, two assumptions are needed;

(i) Conditional independence between cancer and non-cancer mortality.
(ii) Appropriate expected mortality information. This means that the mortality rate due to other causes for the cancer patients is the same as that in the population lifetable.

All methods described below require these assumptions to be true. For example, conditional independence is addressed by stratification, regression modelling or weighting for relevant covariates. For the remainder of this paper we take these assumptions as reasonable as our main focus is on the different methods of estimation.

In summary, net survival can be estimated in a relative survival framework under certain assumptions. However, Eq. (1) is at the individual level and often it is desired to present an average (marginal survival). The traditional lifetable approaches have averaged the numerator and denominator separately, which is not the same as taking an average of individual relative survival and is a key reason why traditional approaches have potential bias when estimating marginal survival.

### Age-standardized relative survival

It is useful to obtain a summary measure of survival in a population. This can be done by calculating the average (or marginal) survival. For simplicity, we consider the situation where relative survival, *R*_*i*_(*t*), only varies by age. An internally age-standardized estimate is the average of the *n* different relative survival curves,

(2) $$  \overline{R}(t) = \frac{1}{n}\sum_{i=1}^{n}{R_{i}(t)},  $$

where *n* is the number of study subjects. This estimates the internally age-standardized net survival, $\overline {S}^{N}\hspace {-2pt}(t)$ under assumptions (i) and (ii). This is not the same as estimating relative survival ignoring the effect of age.

Equation (2) gives the age-standardized relative survival in a particular study population. However, since different populations may have different age distributions, traditional external age-standardization is usually performed. This forces the same age distribution on each population through a weighted average of age-specific estimates of relative survival. The usual external reference population uses weights in five age groups as shown in Table 1 [8]. The externally weighted estimate of age-standardized relative survival adds weights to Eq. (2),

(3) $$  \overline{R}(t) = \frac{1}{n}\sum_{i=1}^{n}{\frac{{w_{i}^{s}}}{a_{i}} R_{i}(t)},  $$

**Table 1:** The three International Cancer Survival Standard (ICSS) weights used for age-standardization of net survival [8]

Age	ICSS 1 ^*a*^	ICSS 2 ^*b*^	ICSS 3 ^*c*^
15-44 years	0.07	0.28	0.60
45-54 years	0.12	0.17	0.10
55-64years	0.23	0.21	0.10
65-74 years	0.29	0.20	0.10
75+ years	0.29	0.14	0.10

^a^Lip, tongue, salivary glands, oral cavity, oropharynx, hypopharynx, head and neck, oesophagus, stomach, small intestine, colon, rectum, liver, biliary tract, pancreas, nasal cavities, larynx, lung, pleura, breast, corpus uteri, ovary, vagina and vulva, penis, bladder, kidney, choroid melanoma, non-Hodgkin lymphomas, multiple myeloma, chronic lymphatic leukaemia, acute myeloid leukaemia, chronic myeloid leukaemia, leukaemia, prostate

^b^Nasopharynx, soft tissues, melanoma, cervix uteri, brain, thyroid gland, bone

^c^Testis, Hodgkin’s disease, acute lymphatic leukaemia

where ${w_{i}^{s}}$ is weight for subject *i* from the reference population (Table 1) and *a*_*i*_ gives the proportion in the age group to which the *i*^*th*^ subject belongs. The ratio, ${w_{i}^{s}}/a_{i}$ will be higher than one in age groups under-represented in the study population compared with the standard population and less than one for age groups over-represented. Since there are only five different weights, in practice relative survival is estimated separately by age group and the weights defined in Table 1 applied, giving,

(4) $$  \overline{R}(t) = \sum_{k=1}^{5}{{w_{k}^{s}} \overline{R}_{k}(t)},  $$

where ${w_{k}^{s}}$ is the weight in the *k*^*th*^ age group and $\overline {R}_{k}(t)$ the corresponding relative survival.

If the weights, ${w_{k}^{s}}$ are replaced by the observed proportion of subjects in each age group then Eq. (4) gives an internally age-standardized estimate, i.e. a grouped version of Eq. (2).

### The Ederer II method

The Ederer II method [18] is estimated in a relative survival framework, i.e. the ratio of all-cause survival, *S*(*t*), to expected survival, *S*^∗^(*t*). The relative survival, *R*(*t*) is,

$$R(t) = \frac{S(t)}{S^{*}(t)} $$

We use an adaption of the Ederer II method and estimate the all-cause and expected survival through back calculation from the all-cause and expected mortality rates. This makes little difference in practice, but allows comparison with the Pohar-Perme method and is similar to the extension of the Ederer II method to continuous time described by Pohar Perme et al. [14].

Follow-up time is divided into a number of intervals and the excess mortality rate, *λ*_*j*_ calculated in each interval by obtaining the total number of deaths in the *j*^*th*^ interval and subtracting the expected number of deaths. Dividing by the total number of person years converts the difference to a rate. For the *j*^*th*^ interval the excess mortality rate is,

$$\lambda_{j}^{E2} = \frac{\sum_{i}{d_{ij}} - \sum_{i}{d_{ij}^{*}}}{\sum_{i}{{y}_{ij}}}, $$

where *d*_*ij*_ is the death indicator for subject *i* in interval *j*, $d_{\textit {ij}}^{*}$ is the expected number of deaths and *y*_*ij*_ is the time at risk. $d_{\textit {ij}}^{*}$ is calculated using $p_{\textit {ij}}^{*}$, the probability individual *i* in interval *j* will survive a year given their attained age and calendar year and other demographic factors,

$$d_{ij}^{*} = -\ln(p_{ij}^{*})y_{ij}. $$

The cumulative excess hazard at the end of interval *j* is,

$$ \Lambda_{j}^{E2} = \sum_{j} k_{j} \lambda_{j}^{E2}, $$

where *k*_*j*_ is the length of the *j*^*th*^ interval. The relative survival is then obtained by,

$$ R_{j}^{E2} = \exp\left(-\Lambda_{j}^{E2}\right) $$

When calculated for the study population as a whole the Ederer II method gives a biased estimate of internally age-standardized net survival unless there is no variation in expected or relative survival between different ages [14, 19], a situation almost certainly not true. Hakulinen *et al.* argued that unless there was substantial variation in relative survival by age the bias would be small, but a theoretical bias still exists [19].

When comparing populations, external age standardization is used with relative survival calculated separately in each age group. Any bias due to variation in relative and expected survival will be reduced. Within each age group the Ederer II method will give an unbiased estimate of the age-specific net survival if there is no variation in relative survival within each age group. This assumption is almost certainly not true, but since narrow age groups are being used the variation will be much reduced compared to the all age estimate. The important question is whether this assumption matters, as empirical observation has shown that the Ederer II method gives a more precise estimate than the Pohar Perme method for long-term estimates [20, 21].

### The Pohar Perme method

The Pohar Perme method was developed to give an unbiased estimate of internally age-standardized net survival [14]. The method estimates the internally age-standardized relative survival, i.e. that defined in Eq. (2). It can be interpreted as internally age standardized net survival under assumptions (i) and (ii). The method gives greater weight to individuals with a higher risk of other cause mortality. For example, consider two individuals aged 60 and 80 at diagnosis. If both are still alive 10 years after diagnosis, the 80 year old receives more weight because similar individuals are more likely to have died of other causes. We use an adaption of the method for when survival time is recorded in monthly intervals [21]. The weight for subject *i* in interval *j* is the inverse of the expected survival, $S_{\textit {ij}}^{*}$, so $w_{\textit {ij}}^{PP} = 1/{S_{\textit {ij}}^{*}}$. The excess mortality rate in the *j*^*th*^ interval is,

$$\lambda_{j}^{PP} = \frac{\sum_{i}{w_{ij}^{PP} d_{ij}} - \sum_{i}{w_{ij}^{PP} d_{ij}^{*}}}{\sum_{i}{w_{ij}^{PP} y_{ij}}}. $$

This is converted to the survival scale in the same way as for Ederer II,

$$\Lambda_{j}^{PP} = \sum_{j} k_{j} \lambda_{j}^{PP} \ \ \ R_{j}^{PP} = \exp\left(-\Lambda_{j}^{PP}\right). $$

For comparisons between population groups external age-standardization is required, so the Pohar-Perme estimate is calculated separately in each age group and a weighted average estimated using Eq. (4).

### Brenner method

It is not possible to calculate traditionally age-standardized relative survival unless all age-specific estimates can be calculated, a problem more likely in the oldest age group. The Brenner alternative method calculates an ‘all age’ Ederer II estimate, with each subject up- or down-weighted so the age distribution reflects the reference population [22]. The weights, ${w_{i}^{B}}$ are the same as those used in Eq. (3).

The excess mortality rate for the *j*^*th*^ interval incorporating these weights is,

$${\lambda_{j}^{B}} = \frac{\sum_{i}{{w_{i}^{B}} d_{ij}} - \sum_{i}{{w_{i}^{B}} d_{ij}^{*}}}{\sum_{i}{{w_{i}^{B}} y_{ij}}} $$

The weights depend on the age distribution at diagnosis. The weighting does not overcome any potential bias if there is substantial variation in relative survival by age. The excess mortality rate is transformed to the relative survival in the same way as above.

### Modelling

There is growing use of statistical models for excess mortality [16, 23]. Modelling has some advantages over non-parametric estimates by allowing more detailed exploration and quantification of differences between groups and can include continuous covariates, such as age at diagnosis[10]. Modelling also allows more precise estimation of parameters of interest by making assumptions. It is also possible to obtain an average or weighted average of individual predicted relative survival functions to obtain internally or externally age-standardized estimates.

Models for excess mortality are of the following form,

$$h_{i}(t) = h_{i}^{*}(t) + \lambda_{i}(t) $$

Interest lies in how covariates affect the excess mortality rate, *λ*_*i*_(*t*). When age-standardizing relative survival only the effect of age needs to be modelled. An internally age-standardized estimate of relative survival is obtained using Eq. (2) where relative survival is estimated for each subject in the study population. An externally age-standardized estimate is calculated using Eq. (3).

We use a flexible parametric survival model for the excess mortality [23]. Through the use of restricted cubic splines the model can capture just about any shape for the baseline excess hazard [24]. It can also incorporate continuous covariates, so the effect of age is captured in more detail, and allow for non-proportional excess hazards. The latter point is important as non-proportional excess hazards is extremely common in these studies and allows the change in the excess mortality rate as a function of age to change as a function of follow-up time.

### Simulation study

The purpose of the simulation study is to quantify any bias in the methods in situations when one would expect the traditional non-parametric lifetable based methods to be biased, i.e. when there is variation in relative survival by age. We have deliberately chosen two scenarios where there is substantial variation by age and, in practice, most cancer sites have far less variation. In addition to quantifying bias we assess the variability in the different estimates.

**Fig. 2:**
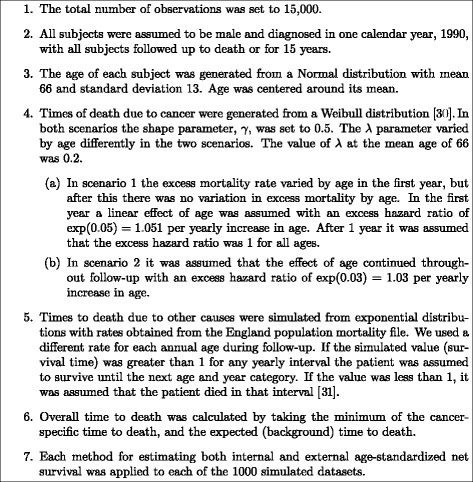
Details of the Simulation Study [30, 31]

Times to death from cancer (net survival) and due to other causes were generated for each individual with the minimum value taken as the time to death. The simulation strategy is outlined in Fig. 2.

Table 2 gives the true net survival at selected ages for each scenario showing large variation by age. The true internally and externally age-standardized estimates are also shown. In the simulation relative survival is equivalent to net survival at the individual level since (i) only age affects relative survival and (ii) the population mortality information used to analyse the data is the same as that used to generate the data.

**Table 2:** True value of net survival for the two scenarios at selected ages at 1, 5, 10, and 15 years postdiagnosis

	Age	1 year	5 years	10 years	15 years
Scenario 1	35	95.8	74.9	62.2	54.0
	45	93.2	72.8	60.5	52.5
	55	89.1	69.6	57.8	50.2
	65	82.7	64.6	53.6	46.5
	75	73.1	57.1	47.4	41.1
	85	59.6	46.6	38.7	33.6
	95	42.6	33.3	27.7	24.0
	Internal	79.7	62.2	51.7	44.9
	External	78.6	61.4	51.0	44.3
Scenario 2	35	92.4	83.8	77.9	73.7
	45	89.9	78.8	71.4	66.2
	55	86.6	72.5	63.5	57.3
	65	82.4	64.8	54.1	47.2
	75	77.0	55.7	43.7	36.3
	85	70.2	45.3	32.7	25.4
	95	62.0	34.4	22.1	15.7
	Internal	81.2	63.3	52.8	46.1
	External	80.6	62.3	51.8	45.1

For each scenario 1000 data sets were generated and estimates of internally and externally age-standardized relative survival obtained using the various methods. These are explained in more detail below.

For internal age-standardization the following methods were used, **Ederer II (all age)** combines all ages into a single group. Time since diagnosis was split into monthly intervals (this is often the detail cancer registries are prepared to release their data). **Ederer II (standardized)** obtains age group specific estimates and uses internal age-standardization using Eq. (4), with the observed proportions in each age group as weights. Time since diagnosis was split into monthly intervals. **Pohar Perme** is calculated for the study population as a whole. Time since diagnosis was split into monthly intervals. **Model-based (grouped)** uses a flexible parametric model with grouped age and applying Eq. (2). The baseline hazard was modelled using 5 df for the spline variables. The effect of age was assumed to be non-proportional by incorporating interactions with follow-up time (3 df for each age group). **Model-based (continuous)** uses a flexible parametric model with continuous age. Restricted cubic splines were used for modelling age (3 df) and applying Eq. (2). The baseline hazard was modelled using 5 df. Non-proportional excess hazards were incorporated through time-dependent effects (3 df for each spline term). Given the way in which the data were simulated, this can be considered over modelling, but it reflects the way models would be applied in practice.

For external age-standardization the following methods were used, **Ederer II (Brenner)** combines all ages and uses the Brenner alternative method to give an externally weighted estimate. **Ederer II (standardized)** obtains age group specific estimates and uses external age-standardization using Eq. (4) using the external reference weights. **Pohar Perme** obtains age group specific estimates and uses external age-standardization using Eq. (4) using the external reference weights. **Model-based (grouped)** uses a flexible parametric model using grouped age and applying Eq. 3. This was the same model as used for internal age-standardization, but applies different weights for the predictions. **Model-based (continuous)** uses a flexible parametric model with continuous age and applying Eq. 3. This was the same model as used for internal age-standardization for the predictions.

Each method was compared to the the true value to give the bias (expressed as difference in percentage points), the mean square error (MSE) and the coverage [25]. The Stata code for the simulations can be found in the online Additional files 1 and 2.

## Results

### Internal and external age-standardization

**Fig. 3:**
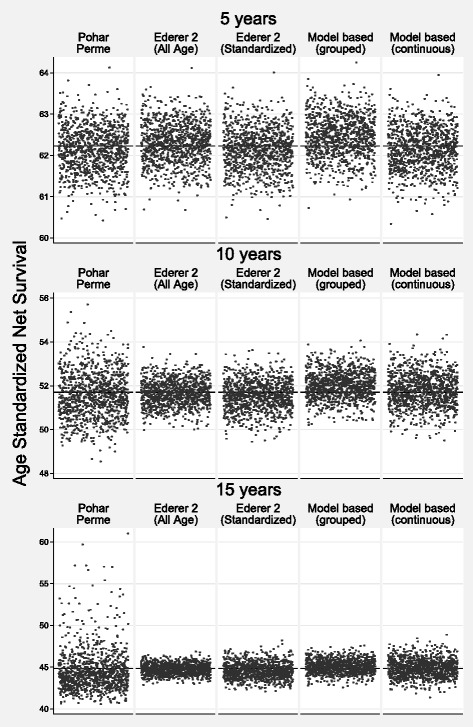
Scenario 1: Scatter Plot of Estimated Internally Age Standardized Net Survival for the 1000 Different Estimates for each of the Methods. The True Value is Shown by a Reference Line

**Fig. 4:**
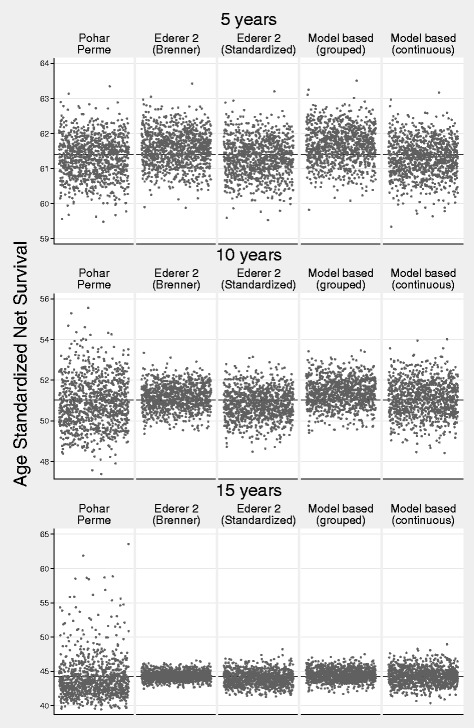
Scenario 1: Scatter Plot of Estimated Externally Age Standardized Net Survival for the 1000 Different Estimates for each of the Methods. The True Value is Shown by a Reference Line

Table 3 shows the bias, MSE and coverage for scenario 1. The bias is low for all methods for both internal and external age standardization. At five years the largest bias was less than 0.27 percentage points (model-based grouped age). Coverage was good for all methods with the exception of the model-based grouped age estimate. With the exception of model-based grouped age, there was more variation in the Pohar-Perme estimate at 5, and particularly 10 and 15 years, reflected in the larger MSEs. This is demonstrated in a scatter plot of the estimates from each simulation in Fig. 3 (internal age standardization) and Fig. 4 (external age standardization). The smallest variation is for the all-age Ederer II method.

**Table 3:** Scenario 1: comparison of bias (bold font), mean square error (normal font) and coverage (italic font) for the different methods to estimate age-standardized net survival. Bias is difference in percentage points

	5 years	10 years	15 years
	Internal age standardization		
Pohar Perme	**-0.0810**	**-0.1266**	**-0.2886**
	0.2746	1.0773	6.2934
	*96.0*	*92.9*	*90.5*
Ederer II	**0.0567**	**-0.0323**	**-0.1005**
(All Age)	0.2344	0.3319	0.4056
	*95.5*	*95.5*	*94.5*
Ederer II	**-0.0883**	**-0.1774**	**-0.2668**
(Standardized)	0.2432	0.4228	0.8489
	*95.0*	*94.0*	*93.1*
Model Based	**0.2208**	**0.3259**	**0.1994**
(Grouped Age)	0.2840	0.4966	0.6919
	*92.7*	*90.3*	*92.5*
Model Based	**-0.0816**	**0.0784**	**0.0278**
(Continuous Age)	0.2394	0.5739	1.1796
	*95.3*	*93.1*	*92.6*
	External age standardization		
Pohar Perme	**-0.0859**	**-0.1292**	**-0.2963**
	0.3109	1.4081	8.7449
	*95.5*	*93.0*	*89.6*
Ederer II	**0.1633**	**0.1372**	**0.1301**
(Brenner)	0.2718	0.3784	0.4702
	*94.8*	*93.7*	*93.0*
Ederer II	**-0.0925**	**-0.1890**	**-0.2849**
(Standardized)	0.2653	0.4934	1.0740
	*95.1*	*93.9*	*93.0*
Model Based	**0.2619**	**0.3655**	**0.2215**
(Grouped Age)	0.3211	0.5756	0.8424
	*92.3*	*90.6*	*93.0*
Model Based	**-0.1017**	**0.0709**	**0.0252**
(Continuous Age)	0.2559	0.6946	1.5279
	*95.3*	*93.3*	*92.6*

**Fig. 5:**
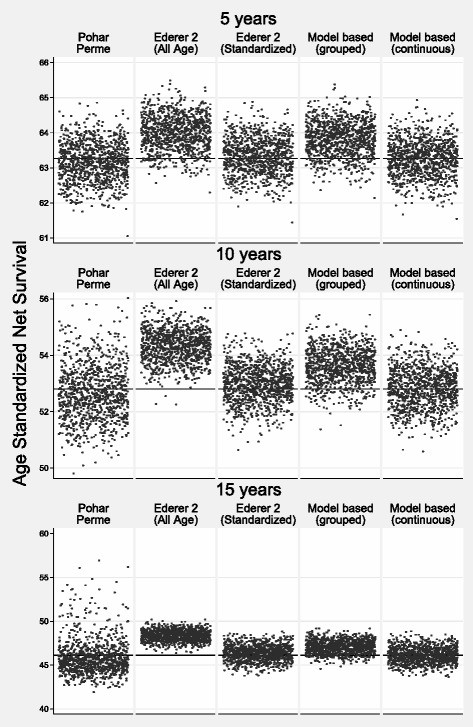
Scenario 2: Scatter Plot of Estimated Internally Age Standardized Net Survival for the 1000 Different Estimates for each of the Methods. The True Value is Shown by a Reference Line

**Fig. 6:**
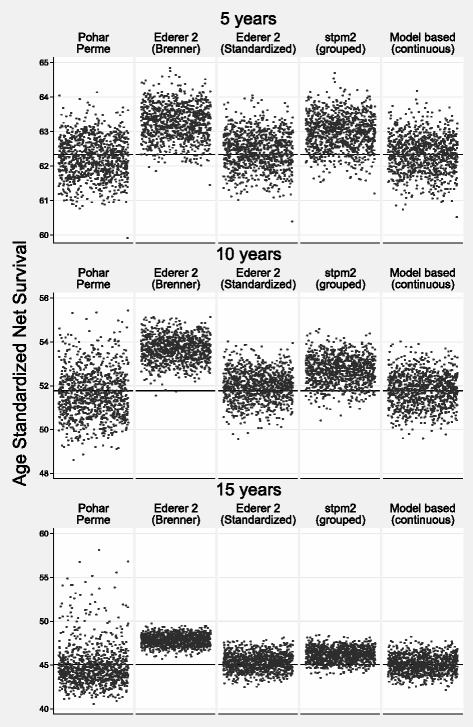
Scenario 2: Scatter Plot of Estimated Externally Age Standardized Net Survival for the 1000 Different Estimates for each of the Methods. The True Value is Shown by a Reference Line

Table 4 shows the bias, MSE and coverage for scenario 2 with Figs. 5 and 6 showing scatter plots of the estimates from each simulation for internal and external standardization respectively. One would expect more bias for the Ederer II method in this scenario since variation by age in excess mortality continues for the whole study follow-up. This can be seen by the fact that the Ederer II all age estimate (internal) and the Brenner method (external) gives biased estimates. For example, a 1.91 and 2.80 percentage point difference for external age standardization for 10 and 15 years respectively. However, the bias for Ederer II using traditional age standardization is less than 0.3 percentage points. There is some bias for the model-based grouped age estimate at a 0.94 and 1.10 percentage point difference for 10 and 15 years respectively. Coverage is reasonable for the Pohar Perme, Ederer II traditional age standardized and model-based continuous age. As for scenario 1 the Pohar Perme method has a higher MSE reflecting more variation in the estimate.

**Table 4:** Scenario 2: comparison of bias (bold font), mean square error (normal font) and coverage (italic font) for the different methods to estimate age-standardized net survival. Bias is difference in percentage points

	5 years	10 years	15 years
	Internal age standardization		
Pohar Perme	**-0.0933**	**-0.1534**	**-0.2379**
	0.2962	0.9117	3.9291
	*95.3*	*95.0*	*91.9*
Ederer II	**0.7287**	**1.5199**	**2.2458**
(All Age)	0.7658	2.6261	5.4354
	*68.0*	*24.0*	*6.1*
Ederer II	**0.0765**	**0.1635**	**0.2229**
(Standardized)	0.2629	0.4573	0.8611
	*95.5*	*94.4*	*94.0*
Model Based	**0.5519**	**0.8263**	**0.9851**
(Grouped Age)	0.5429	1.0404	1.5422
	*78.0*	*71.1*	*73.9*
Model Based	**-0.0008**	**0.0247**	**0.0065**
(Continuous Age)	0.2626	0.4770	0.8395
	*93.9*	*95.0*	*95.6*
	External age standardization		
Pohar Perme	**-0.0999**	**-0.1566**	**-0.2328**
	0.329997	1.155575	5.403732
	*94.8*	*93.8*	*91.1*
Ederer II	**0.9302**	**1.9067**	**2.8049**
(Brenner)	1.108253	3.961526	8.269320
	*53.1*	*10.7*	*1.2*
Ederer II	**0.1027**	**0.2123**	**0.2853**
(Standardized)	0.285740	0.526047	1.053368
	*96.0*	*95.2*	*93.7*
Model Based	**0.6399**	**0.9383**	**1.1026**
(Grouped Age)	0.661305	1.271576	1.886520
	*75.6*	*68.0*	*73.8*
Model Based	**0.0081**	**0.0290**	**0.0074**
(Continuous Age)	0.281334	0.558352	1.049332
	*94.6*	*95.5*	*95.6*

### Age group-specific estimates

To estimate traditionally age standardized net survival separate estimates are required within each age group. For the four youngest age groups there is negligible bias and broad agreement for all methods (data not shown). However, the oldest age group will generally show more bias due to more variation in expected and relative survival in this group. Furthermore, a greater proportion of patients will die from other causes leading to low numbers as follow-up time increases. Table 5 shows the bias, MSE and coverage for the oldest age group for the different methods. Bias for Ederer II for scenario 2 is 0.51, 0.96 and 1.25 percentage points at 5, 10 and 15 years respectively. As with the age standardized estimates, there is far greater variation for the Pohar Perme method, particularly at 10 and 15 years reflected by the higher MSEs.

**Table 5:** Comparison of bias (bold font), mean square error (normal font) and coverage (italic font) for the different methods to estimate age-standardized net survival for those aged 75+. Bias is difference in percentage points

	5 years	10 years	15 years
	Scenario 1		
Pohar Perme	**-0.1590**	**-0.1613**	**-0.6053**
	2.1021	14.3727	101.8314
	*96.2*	*92.5*	*91.4*
Ederer II	**-0.1137**	**-0.3198**	**-0.5318**
	1.5983	3.7299	10.0422
	*96.1*	*95.0*	*95.1*
Model Based	**0.8137**	**0.9559**	**0.5888**
(Grouped Age)	2.0951	3.8231	6.8827
	*90.0*	*92.2*	*94.8*
Model Based	**-0.5241**	**-0.0224**	**0.0451**
(Continuous Age)	1.1701	5.7305	14.7700
	*94.9*	*93.3*	*92.7*
	Scenario 2		
Pohar Perme	**-0.2124**	**-0.2210**	**-0.3109**
	2.4165	11.5840	61.9570
	*95.0*	*94.3*	*92.8*
Ederer II	**0.5060**	**0.9602**	**1.2465**
	1.9939	4.3176	9.9081
	*94.4*	*92.7*	*92.7*
Model Based	**1.9387**	**2.5769**	**2.8401**
(Grouped Age)	5.1811	9.1152	13.3959
	*66.8*	*67.2*	*80.1*
Model Based	**0.1237**	**0.0649**	**-0.0117**
(Continuous Age)	1.6218	4.6727	10.2586
	*94.0*	*95.2*	*94.6*

## Discussion

We have shown through a simulation that when an estimate of age-standardized net survival is required, Ederer II, Pohar Perme and modelling with continuous age have negligible bias. However, Ederer II and modelling have greater precision than the Pohar Perme method, though the increase in precision is small at 5 years.

We do not dispute the theoretical bias in the Ederer II estimate shown by Pohar-Perme [14]. However, when using traditional age-standardization age-group specific estimates are obtained and within each age-group there is far less variation in both expected and relative survival. Thus, even in the extreme scenarios presented here, the potential bias can be ignored. For longer term survival, due to the impact of the weights of elderly subjects, the Pohar Perme estimate has higher variability.

The traditionally age-standardized Ederer II estimate has improved precision over the Pohar Perme estimate since it makes assumptions about variability. The assumptions are there is no variation in expected survival or in relative survival within an age group. The first assumption is definitely not true and the second is unlikely to be. However, these assumptions are not very important in terms of bias. Thus the ‘cost’ of the bias when using Ederer II in traditional age standardization can effectively be ignored, but there is a ‘benefit’ in precision for longer term survival. The difference between the methods is small at five years, but can be seen at 10 and 15 years. For Scenario 1, even the all-age Ederer II had negligible bias due to the variation in age not persisting over follow-up time showing that these assumptions are not that important [19]. The model-based grouped age approach showed some bias, particularly for the more extreme scenario 2. The grouping of age in a statistical model is more prone to bias than when using the Ederer II method due to the different ways of averaging within an age group.

The model-based continuous age approach was unbiased and had improved precision due to making certain assumptions. Firstly, the excess mortality rate is considered a smooth function rather than a step function. The true net survival function can never increase and in practice the estimated relative survival curve when modelling will be a decreasing function of time and not increase due to chance like the Pohar-Perme and Ederer II methods. Secondly, the effect of age is treated as continuous and estimates have greater precision than when grouping age [26]. We used restricted cubic splines to approximate the underlying excess mortality rate as these have been shown to be unbiased in a wide range of scenarios [24]. If interest is only in a single summary measure then we see little advantage of modelling over the non-parametric estimates. However, modelling allows greater understanding of differences between groups of interest. From a single statistical model it is possible to produce summary measures such as age standardized relative survival, but also quantify how differences vary by time since diagnosis, by age or by any other modelled covariate.

Age standardized relative survival is a weighted average of five age groups. The four youngest groups has negligible bias and it is the oldest age group that more likely to be biased, but this group only contributes 29 % of the weight. When interest lies in age-specific estimates the bias may be more important. For example, in our more extreme scenario, the bias for Ederer II for those aged 75+ was around 1 percentage point at 10 and 15 years, though the MSE was substantially lower than the Pohar Perme estimate. This bias could be reduced by using more finely split age groups, but then there would be the potential problem of having no patients at risk for longer term follow-up, so traditional age standardization could not be performed. When modelling age continuously this problem does not occur. An important issue here is whether one should estimate long-term net survival for the elderly. To estimate 15 year net survival in those aged 90, for example, is probably not relevant as it is highly likely that all would be dead by the age of 105. However, if one wants a long-term average estimate for the full population then an estimate in the oldest age group is required. All methods have problems with estimation in the oldest age group. For example, if at diagnosis there are a number of subjects aged 90 or over, but by 4 years these people are all dead. The Pohar Perme estimate has no one over the age of 90 to up weight and thus estimates are based on the survival experience of a younger group. There is a similar problem for the Ederer II and so the younger subjects remaining in the age groups are used for longer term excess mortality estimates. Modelling “borrows” information across both age and follow-up time in the predictions of net survival for older subjects.

Other authors have made strong statements that the Pohar Perme estimate is preferable over Ederer II. However, some of these comparisons have been with the all age Ederer II estimate rather than the traditionally age standardized estimate [14, 15] or placed more emphasis on bias than precision [15]. One paper calculates the difference between the Pohar Perme estimate and various estimates and labels this ‘bias’ [27]. The Pohar Perme estimate is subject to more random variation than the other methods and this is an inappropriate use of the term ‘bias’. A simulation study provides the appropriate framework to quantify bias. One further issue is potential bias when the age distribution changes over calendar time [28, 29]. This can lead to bias, but in most cases this will negligible. Firstly, it is unlikely the age distribution will change as much as in the simulation studies (e.g. a sudden 10 year increase/decrease in the mean age at diagnosis [15]). Secondly, it is not usual to pool so many calendar years together since we would either stratify or model the effect calendar year.

Simulation studies only investigate a limited number of scenarios. Our two scenarios were chosen as very extreme cases due to the variation in net survival by age. The majority of cancer sites will have far less variation. We believe scenario 2 is more extreme than any site seen in practice as the variation in the excess mortality rate by age continues into the long term. Various studies have shown that there is far more variation in the excess mortality rate in the first year or so [9–13, 19]. As the bias is negligible in this extreme case, any potential bias can be ignored in real world applications. Our simulations use a sample size of 15,000. Bias will not be affected by sample size. However, the variation in the Pohar Perme estimate seen in Fig. 1 will become more pronounced in smaller samples.

## Conclusion

In summary, if interest is in a single summary measure of survival we do not see a considerable advantage of the Pohar Perme estimate over the age-standardized Ederer II estimate and a benefit of the latter for longer term follow-up due to improved precision. Our personal preference is to use statistical modelling as it is possible to obtain both simple summaries, such as age standardized estimates, and a more detailed understanding of differences between demographic groups.

## Additional files

Additional file 1
**Stata Do file to generate and analyse the simulated data for scenario 1.** (PDF 3 kb)

Additional file 2
**Stata Do file to generate and analyse the simulated data for scenario 2.** (PDF 3 kb)
